# The ACTIVE cognitive training trial and predicted medical expenditures

**DOI:** 10.1186/1472-6963-9-109

**Published:** 2009-06-29

**Authors:** Fredric D Wolinsky, Henry W Mahncke, Mark Kosinski, Frederick W Unverzagt, David M Smith, Richard N Jones, Anne Stoddard, Sharon L Tennstedt

**Affiliations:** 1 Health Services Research, Iowa City Veterans Affairs Medical Center, Iowa City, Iowa, USA; 2 College of Public Health, University of Iowa, 200 Hawkins Drive, E205 General Hospital, Iowa City, Iowa 52242, USA; 3 Posit Science Corporation, San Francisco, CA, USA; 4 QualityMetric, Inc., Providence, RI, USA; 5 School of Medicine, Indiana University, Indianapolis, IN, USA; 6 Institute for Aging Research, Hebrew Senior-Life, Boston, MA, USA; 7 Aging Studies, New England Research Institutes, Watertown, MA, USA

## Abstract

**Background:**

Health care expenditures for older adults are disproportionately high and increasing at both the individual and population levels. We evaluated the effects of the three cognitive training interventions (memory, reasoning, or speed of processing) in the ACTIVE study on changes in predicted medical care expenditures.

**Methods:**

ACTIVE was a multisite randomized controlled trial of older adults (≥ 65). Five-year follow-up data were available for 1,804 of the 2,802 participants. Propensity score weighting was used to adjust for potential attrition bias. Changes in predicted annualmedical expenditures were calculated at the first and fifth annual follow-up assessments using a new method for translating functional status scores. Multiple linear regression methods were used in this cost-offset analysis.

**Results:**

At one and five years post-training, annual predicted expenditures declinedby $223 (p = .024) and $128 (p = .309), respectively, in the speed of processing treatment group, but there were no statistically significant changes in the memory or reasoning treatment groups compared to the no-contact control group at either period. Statistical adjustment for age, race, education, MMSE scores, ADL and IADL performance scores, EPT scores, chronic condition counts, and the SF-36 PCS and MCS scores at baseline did not alter the one-year ($244; p = .012) or five-year ($143; p = .250) expenditure declines in the speed of processing treatment group.

**Conclusion:**

The speed of processing intervention significantly reduced subsequent annual predicted medical care expenditures at the one-year post-baseline comparison, but annual savings were no longer statistically significant at the five-year post-baseline comparison.

## Background

It is well known that medical expenditures for older adults in the U.S. are disproportionately high, and continue to increase at the individual and population levels [1,2]. A growing literature has shown that the onset of cognitive limitations in older adults, especially as they affect activity of daily living (ADL) and instrumental ADL (IADL) performance, is associated with increased health services use and medical expenditures [3-6]. Based on cross-sectional and longitudinal studies, we have suggested that at the beginning of this process, when cognitive declines are first detected, there is an increase in both physician and hospital use as part of the normal clinical diagnostic and evaluation process [7-10]. But once a dementia diagnosis has been reached, the triage and selection processes are invoked. As a result, those with cognitive limitations choose themselves, or through their families or physicians, to forgo treatment [7-10]. Subsequent work by other investigators has supported our interpretation [11-14].

Based in part on this literature, the National Institutes of Health (NIH) funded the Advanced Cognitive Training for Independent and Vital Elderly (ACTIVE) multisite study. ACTIVE was a randomized controlled trial (RCT) to test the effectiveness and durability of three distinct cognitive interventions in improving proximal outcomes (reasoning, memory, and processing speed), primary outcomes (everyday problem solving, ADLs and IADLs, and everyday speed), and secondary outcomes (health-related quality of life [HRQoL], health services use, and health care expenditures) [15].

Although all three ACTIVE treatments have been shown to be effective at improving their targeted abilities (proximal outcomes) at post-test, the speed of processing training group demonstrated the largest gains, with 87% of participants showing reliable improvement after the intervention [16]. Furthermore, while each intervention continued to show improvements in their targeted cognitive abilities relative to baseline at both two- and five-year follow-ups the greatest relative improvements in targeted abilities were clearly associated with the speed of processing group [16,17]. Indeed, the effect sizes at all time points for the speed of processing group were more than double those associated with the other interventions.

Statistically and clinically significant effects of ACTIVE's speed of processing intervention have also been shown on many of the secondary or health outcomes, relative to the no-contact control group, including: (1) a 38% reduction in the risk of global decline in HRQoL at two-years post-baseline (*p *= .004), and a 25.6% reduction in the risk of global decline in HRQoL (*p *< .038) at five-years post-baseline [18,19]; (2) a 30% reduction in the risk of worsening depressive symptoms at both one-year (*p *= .012) and five-years (*p *= .023) post-baseline [20]; (3) a 38% reduction in the risk of the onset of suspected clinical depression at one-year (*p *< .01) post baseline [21]; (4) improvements in self-rated health at two-, three-, and five-years equivalent to at least half of the difference between "excellent" and "very good" responses (*p *values < .05), which is known to be associated with a 0.8% absolutereduction in the five-year mortality rate, and a 10% relative mortality reduction (Wolinsky FD, Mahncke HW, Vander Weg MW, Martin R, Unverzagt FW, Ball KK, Jones R, Tennstedt SL, Speed of processing training improves self-rated health in older adults: enduring effects observed in the multi-site ACTIVE study, submitted); and, (5) a 64% greater likelihood (*p *< .05) of improvements in internal locus of control at five-years post-baseline (Wolinsky FD, Vander Weg MW, Martin R, Unverzagt FW, Willis SL, Marsiske M, Rebok GW, Morris JN, Ball KK, Tennstedt SL, Cognitive training improves internal locus of control among older adults, submitted).

In this article, we extend the focus on secondary outcomes by translating patient-reported outcomes into meaningful variations in cost estimates appropriate for consideration by diverse health care delivery stakeholders. Ideally, this would be done using actual Medicare and other payor claims. At the present time, however, we do not have access to Medicare claims for the entire follow-up period, nor to other payor claims at all. Therefore, we use knowledge from empirical data showing the relationship between functional status and medical expenditures to translate changes over time in SF-36 composite scores into predicted annual medical expenditure savings [22].

## Methods

### Design

A detailed description of ACTIVE is available elsewhere [15]. Here we present sufficient information for background purposes. ACTIVE was a multisite, single-blind RCT with three treatment arms and a no-contact control group. It was hypothesized that each of the three intervention arms would have a direct effect on its targeted, trained outcome (proximal outcomes), and nonspecific effects on each of its non-targeted, untrained outcomes. It was further hypothesized that the effects of the ACTIVE interventions on both the primary and secondary outcomes would be mediated through the targeted, trained (proximal) outcomes. Among the primary outcomes, the reasoning and memory interventions were expected to affect only everyday problem solving and ADLs and IADLs, whereas the speed of processing intervention was hypothesized to have more diverse effects, including ADLs and IADLs, everyday speed, and driving habits. All three ACTIVE interventions were expected to affect the secondary outcomes, including HRQoL, mobility, health services use and expenditures.

### The Original ACTIVE Sample

Although all ACTIVE study participants were at risk for loss of functional independence, they had to live in the community independent of formal care and be 65 years old or older. Each of six sites used slightly different recruitment strategies to identify 4,970 potential participants from March 1998 through October 1999 [15]. Of these, 905 (18.1%) were excluded if they demonstrated cognitive impairment (a Mini-Mental Status Examination [MMSE; 23] score < 23), had corrected vision less than 20/50, were dependent in hygiene, bathing or dressing, had ever been diagnosed as having Alzheimer's Disease or had had a stroke during the prior year, reported limited life expectancy due to cancer or were undergoing chemotherapy or radiation treatment at baseline, had difficulty communicating, planned on moving within one year, anticipated having scheduling conflicts, or had previously undergone cognitive training. Another 1,263 potential participants (25.3%) refused to participate in either the screening or enrollment processes. The 2,802 remaining potential participants were screened, enrolled, and randomized.

### Interventions

Each of the three interventions involved ten sessions that shared key elements, and involved 1-hour sessions. The ten sessions were spread over six weeks, with an average group size of 3–4 participants per group. Strategy instruction and practice exercises were the focus of the first five intervention sessions, while the last five provided additional practice. Both the laboratory-type and everyday activities used were well-specified in trainer manuals, and the intervention trainers underwent extensive training, certification, and continuous quality improvement evaluation and review [15]. The focus of the *reasoning training *was on inductive reasoning, especially the ability to solve problems that followed a serial pattern and were manifest in executive functioning. The focus of the *memory training *was on verbal episodic memory, especially using mnemonic strategies for remembering lists, sequences of items, text material, and main ideas and story details. The focus of the *speed training *was on visual search and the ability to identify and locate visual information in a divided attention format.

### Booster Sessions

About one month prior to the first and third annual follow-ups, booster training was offered to a 60% random sample of cognitive intervention participants who had completed at least 80% of the initial training sessions. These participants received up to four additional standardized sessions at each of those two follow-ups under equivalent circumstances. Because the method of selecting participants to receive booster training was conditioned (i.e., dependent) on participant adherence, we do not consider the booster effect in our analyses. That is, we estimate a pooled effect within each of the three intervention groups that reflects both those who were and were not invited to participate in the booster sessions. The assumption underlying this approach is that all trained participants received the booster training, and that there was no effect of booster training in those not actually receiving booster training. This assumption biases our results towards the null, and is overly conservative because 89% of persons who were not randomized to booster training were eligible for booster training. Eligibility for booster training did not significantly differ by training group.

### Predicted Medical Expenditures

We have previously shown significant effects of the cognitive interventions on clinically important differences in HRQoL with the widely used eight SF-36 scales [24-30] at both two and five years post-training [18,19]. Here we use a method recently developed by an investigative team from the Agency for Healthcare Research and Quality (AHRQ), the University of Chicago, and QualityMetric [22] for expressing observed changes in the overall Physical Composite Scores (PCS) and Mental Composite Scores (MCS) of either the SF-12 or the SF-36 into changes in predicted annual medical expenditures from baseline to the one-year follow-up, and from the third-year to the fifth-year follow-up. Detailed descriptions and procedures for using and scoring the SF-36, and the exact wording of the SF-36 items are available elsewhere [24-30]. Scores on the PCS and MCS theoretically range from 0 (worst health) to 100 (best health).

This new method was developed and validated using 5,542 participants from the 2000–2001 population-based, nationally-representative Medical Expenditure Panel Study (MEPS) sponsored by AHRQ to chart expenditure trends in the US. In their new approach, Fleishman et al. used SF-12 scores to predict mean monthly expenditures over the next year that were obtained from linked administrative claims data [22]. Using Poisson regression, their most sophisticated model (Model 6 in their Table Four [22]) included age, gender, demographics, medical conditions, the PCS and MCS scores, and prior health expenditures; it explained 29.2% of the variance in actual medical expenditures, which is relatively robust in the expenditures literature [20]. Because unlike MEPS, ACTIVE was an RCT, we begin with Fleishman et al.'s base model (Model 1 in their Table Four [22]) which only includes age, gender, and the PCS and MCS scores, and explained 13.4% of the variance in actual medical expenditures. Our justification is that in the ACTIVE RCT we may expect, and have previously shown, equivalence on nearly all observed factors across treatment groups at baseline [16-19]).

Because all ACTIVE participants were 65 years old or older at baseline, the equation for predicted annual medical care expenditure then becomes:



where *male *and *age65 *are binary markers for being a man (vs. a woman) and being 65 years old or older (vs. being younger), and *male_age65 *is the simple multiplicative interaction term reflecting older men. The exponent (of the bracketed, i.e., [x]) value is taken to yield dollar values, because the coefficients shown are the un-exponentiated Poisson regression coefficients obtained by Fleishman et al [22]. The exponentiated value is then multiplied by 12 to obtain the estimate of predicted annual medical expenditures, because the Fleishman et al.'s original equation was for monthly expenditures [22]. Finally, to obtain a differences in differences (or change) analysis, we subtracted the annual expenditure estimate obtained at baseline from the annual expenditure estimate obtained at the one-year follow-up, and we subtracted the annual expenditure estimate obtained at the three-year follow up (because there was no four-year follow-up) from the annual expenditure estimate obtained at the five-year follow-up.

As an added safeguard to the analysis of the base model described above, we used multiple linear regression to statistically adjust the effects of treatment group first for age, race, education, MMSE scores [23], ADL performance [31], IADL performance [32], everyday cognitive performance scores [33], and medical conditions [34], and then for baseline PCS and MCS scores [29,30] as well. Thus, our final analysis considers all of the factors in Fleishman et al.'s final model (i.e., Model 6 in their Table Four [24]) except for prior observed expenditures, which we do not have in ACTIVE.

### Analytic Sample

Of the 2,802 participants who were screened, enrolled, and randomized, 1,804 (64.4%) were successfully reassessed on all outcomes at the fifth annual follow-up. We restrict our analysis to these 1,804 participants, regardless of group assignment, treatment adherence, or booster status, for two reasons. First, doing so maintains complete comparability to our prior reports [19]. Second, this approach avoids compositional change issues in comparing the one-year and five-year results. Attrition was not associated with treatment status [18,19].

### Attrition Bias

Our focus on the 1,804 (64.4%) of the 2,802 original ACTIVE participants who were reassessed on the HRQoL outcomes at the five-year follow-up raises the potential for attrition bias in relation to outcomes of interest. Therefore, as in our prior reports, propensity score methods were used to adjust for potential attrition bias [35-37]. We estimated a multivariable logistic regression model of whether outcome data were available at the five-year follow-up, and computed the predicted probabilities of inclusion in the analytic sample. The propensity score model has previously been reported [19]. This model included binary indicators for each of the three cognitive intervention arms, and baseline age, sex, race, MMSE scores, ADLs, IADLs, EPT scores, depressive symptoms, comorbid medical conditions, and SF-36 scores. We then determined the average participation rate (i.e., whether five-year follow-up data were available, or P) within each propensity score (predicted probability) quintile, and used the inverse (1/P) to weight the data. This gives greater influence to retained participants who were most like those not followed. Finally, the propensity score weights were adjusted so that the weighted N was 1,804 (equal to the number of participants actually reassessed at the five year follow-up).

## Results

### Descriptive

Table 1 contains the unadjusted or crude means or percentages for the variables of interest in the analytic sample using the propensity score weighted data overall, and by treatment group. Overall, at baseline the mean age was 75.7, 25% were men, 31% were Black, and the average educational attainment was 13.4 years. The mean MMSE [23] score was 27.2, the average score on the Minimum Data Set (MDS) ADL performance scale [31] was 0.3 (observed range = 0 to 10), the average score on the MDS IADL performance scale [32] was 4.2 (observed range = 0 to 23), the average score on the EPT [33] was 18.3, and the mean number of chronic conditions [34] was 2.3. At baseline, the mean PCS score was 42.3, the mean MCS score was 53.6, and the average predicted annual medical expenditure was $6,741. The only statistically significant difference across treatment groups in these baseline variables involved the MCS score, which ranged from a low of 52.7 in the reasoning group to a high of 54.4 in the no-contact control group. From baseline to the first annual follow-up the speed of processing group showed a mean MCS score improvement (0.982), although this was marginally insignificant (p = .100). From the third to the fifth annual follow-up the speed of processing group again showed a mean MCS score improvement (0.701) that was statistically significant (p = .009). Note that the comparisons shown in Table 1, however, are not adjusted for any of the covariates shown in that table.

**Table 1:** Unadjusted Baseline and Follow-Up Means or Percentages among the ACTIVE Participants (Weighted N = 1,804) in the Analytic Sample, by Treatment Group.

**Variable**	**Memory Group (N = 444)**	**Reasoning Group (N = 447)**	**Speed of Processing Group (N = 452)**	**Control Group (N = 462)**	**Total**
*Baseline*					
Age (years)	75.8	76.0	75.2	75.9	75.7
**Men (%)**	24.8	26.3	24.8	25.3	25.3
**Black (%)**	29.1	30.4	33.4	32.3	31.4
**Education (years)**	13.5	13.3	13.5	13.4	13.4
**MMSE (score)**	27.2	27.1	27.2	27.2	27.2
ADL Count*	0.42	0.31	0.26	0.31	0.32
**IADL Count**	4.16	4.26	4.11	4.16	4.17
**EPT Score**	18.67	17.98	18.35	18.31	18.33
**Medical Cond. Count**	2.3	2.4	2.4	2.2	2.3
**PCS Score**	42.68	41.16	42.93	42.31	42.27
MCS Score*	54.26	52.71	53.22	54.38	53.65
**Predicted Annual Medical Expenditures**	6,469	7,163	6,576	6,756	6,741
***Changes in SF-36 Composite Scores***					
**PCS (baseline to 1 year)**	-.556	-.452	-.129	-.450	-.399
**MCS (baseline to 1 year)**	-.408	-.033	.982	.314	.208
PCS (3 to 5 years)**	-.313	.221	-.680	.168	-.151
MCS (3 to 5 years)**	.224	-.648	.701	-1.198	-.231
***Predicted Annual Medical Expenditure Changes***					
Baseline to 1 year*	146.65	199.31	-130.59	91.96	78.34
**3 to 5 years**	229.12	234.90	73.99	202.00	184.76

Notes: The analytic sample was restricted to ACTIVE participants successfully re-interviewed at the fifth annual follow-up, with propensity score weighting used to adjust for potential attrition bias, in order to avoid compositional incomparability to our prior report [19]. MMSE = mini-mental status exam; ADL = activities of daily living; IADL = instrumental ADLs; EPT = everyday performance test; and, PCS = physical component score; MCS = mental component score.

* = p < .05; ** = p < .01

### Multiple Linear Regression

In contrast to the baseline findings shown in Table 1, both predicted annual medical expenses at the first annual follow-up as well as the change in those predicted expenditures since baseline were statistically significantly different across the treatment groups. Overall, predicted annual medical expenses rose from baseline to the first annual follow-up by $78.34, resulting in annual predicted expenses of $6,929. But there was considerable variation across treatment groups. Tables 2 and 3, contain the partial, unstandardized (B) coefficients obtained from the three-step multiple linear regression of the changes in predicted annual medical expenditures clarifies the pattern in the variation. Note that the reference group in all models shown in Tables 2 (baseline to first annual follow-up) and 3 (third annual to fifth annual follow-ups) is the no-contact control group.

**Table 2:** Partial, Unstandardized (B) Coefficients (Dollar Values) Obtained from Three-Step Multiple Linear Regression Models of the Changes in Predicted Annual Medical Expenditures at the One-Year Annual Follow-Up among the ACTIVE Participants (Weighted N = 1,804) in the Analytic Sample.

Treatment Group	Change in Expenditures at the First Annual Follow-up	Change in Expenditures at the First Annual Follow-up	Change in Expenditures at the First Annual Follow-up
	Step One	Step Two	Step Three

Memory	54.70	80.66	69.29

Reasoning	107.35	121.23	121.22

Speed of Processing	-222.55*	-215.49*	-243.99*

Notes: The analytic sample was restricted to ACTIVE participants successfully re-interviewed at the fifth annual follow-up, with propensity score weighting used to adjust for potential attrition bias, in order to avoid compositional incomparability to our prior report [19]. Step one includes the three treatment group variables. Step two includes the three treatment group variables, and age, race, education, MMSE, ADLs, IADLs, EPT scores, and medical conditions. Step three includes the three treatment group variables, age, race, education, MMSE, ADLs, IADLs, EPT scores, and medical conditions, as well as the baseline PCS and MCS scores. The control group is the omitted or reference category.

* = p < .05

**Table 3:** Partial, Unstandardized (B) Coefficients (Dollar Values) Obtained from Three-Step Multiple Linear Regression Models of the Changes in Predicted Annual Medical Expenditures at the Five-Year Annual Follow-Up among the ACTIVE Participants (Weighted N = 1,804) in the Analytic Sample.

Treatment Group	Change in Expenditures at the Fifth Annual Follow-up	Change in Expenditures at the Fifth Annual Follow-up	Change in Expenditures at the Fifth Annual Follow-up
	Step One	Step Two	Step Three

Memory	27.12	58.66	44.58

Reasoning	32.90	23.99	42.75

Speed of Processing	-128.01	-123.96	-143.02

Notes: The analytic sample was restricted to ACTIVE participants successfully re-interviewed at the fifth annual follow-up, with propensity score weighting used to adjust for potential attrition bias, in order to avoid compositional incomparability to our prior report [19]. Step one includes the three treatment group variables. Step two includes the three treatment group variables, and age, race, education, MMSE, ADLs, IADLs, EPT scores, and medical conditions. Step three includes the three treatment group variables, age, race, education, MMSE, ADLs, IADLs, EPT scores, and medical conditions, as well as the baseline PCS and MCS scores. The control group is the omitted or reference category.

* = p < .05

The only statistically significant effects are shown in Table 2 for the one-year follow-up was for the speed of processing treatment group. In terms of changes in predicted expenditures, these data show that there was a statistically significant decline in annual predicted medical expenditures from baseline to the first annual follow-up of $222.55 (p = .024) for the speed of processing group, and that further statistical adjustment for age, race, education, MMSE scores, ADLs, IADLs, EPT scores, and medical conditions (i.e, step two), as well as the baseline PCS and MCS scores (i.e., step three) did not alter this effect. The effects in the memory and reasoning treatment groups were not statistically significant in this comparison. As shown in Table 3, from the third annual follow-up to the fifth annual follow-up, although the decline in annual predicted medical expenditures for the speed of processing group continued, it was notably smaller and no longer statistically significant ($128.01; p = .309). Again, the effects in the memory and reasoning treatment groups were not statistically significant in this comparison.

## Discussion

### Summary

Our cost-offset analyses have shown significant differences by treatment intervention group in annual predicted medical expenditures from baseline to the one-year follow-up. Those differences were driven by the lower predicted expenditures observed among participants in the speed of processing treatment group. When compared to the control group, those differences amounted to predicted annual savings of $223 or $215 or $244, depending on whether the treatment effects were unadjusted, or adjusted for age, race, education, MMSE scores, ADLs, IADLs, and the EPT scores, or adjusted for those factors as well as the baseline PCS and MCS scores, respectively. By the end of the five year follow-up period, however, the annual difference in predicted medical expenditures had notably diminished and was no longer statistically significant.

### Importance

These results are especially important for three reasons. First, ACTIVE is the largest multisite RCT ever conducted that focused on improving or maintaining cognitive performance among older adults [15], which enhances both ACTIVE's internal and external validity. Second, the predicted expenditures in the ACTIVE sample are reasonably representative of the Medicare population. This is reflected in the fact that the first annual follow-up per capita predicted annual expenditures for ACTIVE ($6,929) were comparable to the national per capita base (i.e., age ≥ 65) average annual estimate for older adults in 2003 under the applicable Medicare Advantage (MA) capitation rate structure ($6,638) [38]. Finally, the speed of processing intervention is computer-based, designed to be self-administered, and could allow participants to proceed at her/his own pace, thus increasing the likelihood that maximal effective dosing is delivered. At the same time, however, our results are not particularly surprising, because the estimated expenditures are ultimately just a retransformation of the original SF-36 data which we had used in prior analyses [18,19]. That said, our approach does provide meaningful variations in cost estimates appropriate for consideration by diverse health care delivery stakeholders.

### Limitations

Our study is not without limitations. The most important of these is our reliance on the new method [22] to predict annual medical expenditures. Under ideal circumstances, we would involve used actual administrative claims data files. Unfortunately, we do not have Medicare claims for the entire follow-up period, and we do not have any other payor claims at all. Particularly relevant in this population are costs for services such as homemaker and meals that enable and support independent living for which claims are not available. Accordingly, further research that examines actual expenditure savings to Medicare and other payors over this period is necessary to verify the predicted expenditure savings reported here. It is also important to note that we did not use a smearing estimator [39,40] in the process of generating the predicted expenditure estimates [22], and as a result, our standard errors may have been underestimated after exponentiation of the logged estimates to transform them back into dollars. In sensitivity analyses (not shown), however, we replicated our analyses using the logged estimates themselves, and found robust results with equivalent effect sizes and significance levels. Thus, it is unlikely that underestimation of the standard errors is problematic.

### Policy Relevance

Our results have significant health policy relevance. The speed of processing intervention was able to reduce predicted medical expenditures by 3.2% ($223/$6,929) between baseline and the first annual follow-up. Moreover, we emphasize here the fact that ACTIVE participants were only allowed ten 1-hour training sessions at baseline, unless they had been randomized, conditioned upon completing at least 8 of the ten baseline intervention sessions, to receive up to four additional standardized sessions one-month prior to the first and third annual follow-ups. Because the receipt of booster training was conditioned on participant adherence, however, we cannot address the "dosing" question (i.e., the separation of the basic intervention effect [up to 10 hours] from the booster effect [up to 8 additional hours for those so randomized]) in an intent-to-treat format. Nonetheless, when we have explored the "dosing" issue from an effectiveness standpoint for other outcomes, the results have been what one would expect–greater effects for those randomized to basic and booster speed of processing training, than for those randomized to just basic speed of processing (Wolinsky FD, et al., Speed of processing training improves self-rated health in older adults: enduring effects observed in the multi-site ACTIVE study, submitted; (Wolinsky FD, et al., Cognitive training improves internal locus of control among older adults, submitted).

### How Speed of Processing Works

At this point, it is important to raise, if not address, the ultimate question–how did the speed of processing intervention reduce predicted expenditures? As we have noted before, speed of processing operates through sensory-motor elaboration and repetition [15-19], and procedural tasks have a broader pattern of regional brain activation than explicit memory tasks [41]. We believe that the resulting improvements in brain activation and/or structure delayed the onset or reduced the risk of cognitive slowing, which has been argued to be among the most significant contributors to overall cognitive decline [42,43].

That said, we conducted additional (ad hoc) analyses to determine whether the effect of speed of processing on predicted medical expenditures was direct, indirect, or both. First, we calculated the baseline to one-year follow-up improvement in processing speed. In our analytic sample overall, there was an average improvement (reduction) in processing time on the Useful Field of Vision (UFOV) test [15-17] of 156 milliseconds (ms; standard deviation = 211 ms), with the improvements in the memory, reasoning, and no-contact control groups ranging from 98 to 101 ms, vs. an average improvement in the speed of processing group of 321 ms (p < .0001). Thus, random assignment to the speed of processing intervention resulted in a net UFOV test improvement of about 220 ms more than any other group, a differential effect size of about 1.0. This was expected, because the UFOV test was the proximal target outcome for the speed of processing intervention.

We then added the one-year improvement in the UFOV test to the model reflected in column two (i.e., change in expenditures at the first annual follow-up) of Table 2. Doing so did not appreciably alter the effects for the memory or reasoning groups, which remained statistically insignificant (p > .40). The effect for the speed of processing group, however, increased to a $295.39 medical expenditure reduction (p = .016), and the effect for UFOV test improvement was a $0.44 lower medical expenditure per ms of improvement in processing speed (p = .033). The standardized regression coefficients for these effects were fundamentally equivalent (i.e., -0.089 vs. -0.067, respectively). On the one hand, this indicates that random assignment to the speed of processing intervention group reduced predicted medical care expenditures, and that the greater the improvement in processing speed, the greater the reduction in predicted medical expenses. To the best of our knowledge, this is the first demonstration that improvements in processing speed transfer to distal health outcomes. On the other hand, it indicates that the etiologic mechanism here is not just direct, because for that to have been the case, the effect of random assignment to the speed of processing group should have been dramatically reduced and no longer statistically significant. Thus, speed of processing has both direct and indirect effects on predicted medical expenditures. Further research will be needed to identify the causal pathways involved in those indirect effects.

## Conclusion

The speed of processing intervention significantly reduced subsequent annual predicted medical care expenditures at the one-year post-baseline comparison, but annual savings were no longer statistically significant at the five-year post-baseline comparison. This is not surprising because the 1-year follow-up analysis would best showcase the potential effects of the ACTIVE cognitive interventions on predicted medical expenditures. The reason is that each of the three ACTIVE interventions involved a rather low dose and rather short duration, and therefore their effects would be most observable at 1-year post-baseline.

## Competing interests

Dr. Wolinsky's efforts on the analysis for and writing of this article were supported in part by a limited consulting arrangement with Posit Science Corporation, of which Dr. Mahncke is Vice President for Research and Outcomes, and a stock holder. Posit Science Corporation acquired ownership of the speed of processing intervention used in the ACTIVE Cognitive Training Trial in October 2007. The authors declare that there are no other competing interests.

## Authors' contributions

FDW designed and conducted all of the analyses, interpreted the results, and drafted and revised the manuscript. HWM and MK conceived of applying the predicted expenditures algorithm to the ACTIVE data, and MK is a co-developer that algorithm. AS provided oversight of the statistical analysis. FDW, FWU, DMS, RNJ, and SLT participated in the conceptualization of the ACTIVE grant applications and the overall study design, and provided substantive expertise. All authors read and approved the final manuscript.

## Pre-publication history

The pre-publication history for this paper can be accessed here:


